# Ameliorative effects of *Cirsium japonicum* extract and main component cirsimaritin in mice model of high‐fat diet‐induced metabolic dysfunction‐associated fatty liver disease

**DOI:** 10.1002/fsn3.2548

**Published:** 2021-09-01

**Authors:** Denis Nchang Che, Jae Young Shin, Hyun Ju Kang, Byoung Ok Cho, Ji Hyeon Park, Feng Wang, Suping Hao, Jae Suk Sim, Dong Jun Sim, Seon Il Jang

**Affiliations:** ^1^ Institute of Health Science Jeonju University Jeonju‐si Republic of Korea; ^2^ Department of Food Science and Technology Jeonbuk National University Jeonju‐si Republic of Korea; ^3^ Department of Environmental Science and Biotechnology Jeonju University Jeonju‐si Republic of Korea; ^4^ Research Institute Imsil Herbal Medicine Association Imsil Republic of Korea; ^5^ Department of Health Management Jeonju University Jeonju‐si Republic of Korea

**Keywords:** Cirsimaritin, *Cirsium japonicum*, fatty liver, inflammation, MAFLD, oxidative stress

## Abstract

The objective of this study was to determine biological effects of *Cirsium japonicum* extract and its main component cirsimaritin on high‐fat diet (HFD)‐induced metabolic dysfunction‐associated fatty liver disease (MAFLD) in a mouse model. Mice were fed with a HFD to induce MAFLD and simultaneously administered with *C*. *japonicum* extract (CJE) or cirsimaritin. Various MAFLD biomarkers were evaluated using biological methods. Results demonstrated that triglyceride, aspartate aminotransferase, alanine aminotransferase, and malondialdehyde levels in the liver of mice were significantly reduced upon administration of CJE or cirsimaritin. Treatment with CJE or cirsimaritin also reduced the severity of liver injury in the experimental mouse model of MAFLD by inhibiting hepatic steatosis, oxidative stress, inflammation, and liver fibrosis. These results demonstrate that CJE and cirsimaritin as its main compound have a preventive action against the progression of hepatic steatosis to fibrosis and cirrhosis. Our study suggests that CJE and cirsimaritin might be promising agents for preventing and/or treating MAFLD.

## Graphical abstract

*Cirsium japonicum* and Cir improved HFD‐induced MAFLD through inhibition of hepatic steatosis, oxidative stress, and inflammation.

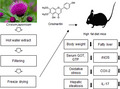

## 1 INTRODUCTION

Metabolic dysfunction‐associated fatty liver disease (MAFLD) is one outcome of obesity, a dreaded disease that is classified by WHO as an epidemic. It is a disease of public health importance in Europe and other Western countries partly due to its complicated pathology and the lack of effective prevention and treatment methods (Angulo, 2002; Cohen et al., 2011; Hou et al., 2011; Levene & Goldin, 2012). It is also one of the most common liver disorders associated with obesity, diabetes, and insulin resistance (Levene & Goldin, 2012). Specifically, MAFLD corresponds to a group of pathologies ranging from simple fatty liver (steatosis) to more severe nonalcoholic steatohepatitis (NASH), liver fibrosis, cirrhosis, and hepatocellular carcinoma (HCC). As the name implies, it is characterized by excessive fatty acid infiltration into hepatocytes in the absence of excessive alcohol consumption (Targher et al., 2016). Up to date, the exact etiology and pathology of the disease are still unclear. However, the accumulation of fatty acid in the liver caused by abnormal regulation of free fatty acid absorption, fatty acid synthesis, and fatty acid oxidation is evidenced (Periasamy et al., 2014). This disruption of the metabolic processes in the liver exposes the liver to oxidative stress that results in inflammation and insulin resistance (George et al., 2003; Povero et al., 2010; Sanyal et al., 2001). The association of inflammation in MAFLD has been recently confirmed as IL‐17, a pro‐inflammatory cytokine was shown to be involved in the development of alcoholic fatty liver disease, viral hepatitis, cholestatic cirrhosis, and other chronic liver diseases (Lafdil et al., 2010; Tan et al., 2013). Not only is IL‐17 involved in alcoholic fatty liver disease, but has also been demonstrated to promote nonalcoholic fatty liver and nonalcoholic steatohepatitis, thus indicating that IL‐17 may contribute to MAFLD progression (Harley et al., 2014). In addition, neutralization of IL‐17 by anti‐IL‐17mAb significantly improved liver function, attenuated hepatic lipid accumulation, suppressed Kupffer cells activation and decreased pro‐inflammatory cytokines levels in mice fed with high‐fat diet (Xu et al., 2013). These findings provide strong evidence to support research in the development of drugs that could prevent IL‐17 production and hence prevent MAFLD.

Although lifestyle (diet and exercise) management is one way to prevent the progression of MAFLD, the best strategy for managing MAFLD has not been defined yet. Natural products have provided basis for the development of potent synthetic drugs in the past. They have a long history of being used to reduce health risks and improve health conditions. It has been shown that natural products and their phenolic compounds can diminish inflammation and HFD‐induced liver diseases in both in vitro and in vivo studies (Choi et al., 2017; Li et al., 2020). Among various natural products, *Cirsium japonicum,* listed in Korean and Chinese pharmacopeias has been used in Chinese and Korean traditional medicine for treating hemorrhagic fever, hypertensive, hepatitis, and urinary track diseases (Yeon Park et al., 2017). It is a member of a perennial herbaceous species found in Korea, China, and Japan. It contains many medicinal components including cirsimaritin. *C*. *japonicum* extract and cirsimaritin were found to induce apoptosis and prevent proliferation of human breast cancer cells (Kim et al., 2010; Yeon Park et al., 2017). The extract, and one of its most potent constituents, apigenin, was also shown to block Hif‐2α‐induced osteoarthritic cartilage destruction (Cho et al., 2019). Although various pharmacological activities of this extract have been reported, the effects of *C*. *japonicum* extract and cirsimaritin on MAFLD have not been studied. The objective of this study was to determine inhibitory effects of *C*. *japonicum* extract and cirsimaritin on high‐fat diet (HFD)‐induced MAFLD in a mouse model.

## 2 MATERIALS AND METHODS

### 2.1 Plant extract preparations

The plant extract was obtained using a hot water extraction method. Briefly, 100 g (dry weight) of *C*. *japonicum* was extracted with 1.5 L of distilled water for 8 h at 105℃. After extraction, the extract (hereafter named CJE) was filtered using a Whatman filter paper (ADVANTEC, Togo, Japan), concentrated using a Vacuum Rotary Evaporator, and freeze‐dried to obtain the powder form of CJE. Milk thistle (MT) was obtained as a commercial dietary supplement from GNC Herbal Plus, Seoul, Korea. Cirsimaritin was purified from the extract by the Natural Product Institute of Science and Technology (NIST), Korea.

### 2.2 Animal experiments

Fifty‐five adult male C57BL/6 mice (8 weeks of age) with an average weight of 23 ± 1 g were purchased from Orient Bio Inc. (Gwangju, Republic of Korea) and used in this study. The mice were housed under pathogen‐free conditions in the animal facility of Jeonju University. They were fed for 8 weeks with either a high‐fat (HF) diet which provided 59% of calories from fat, 25% of calories from carbohydrates, and 16% of calories from proteins or a normal diet (ND), which provided 12% of calories from fat, 59% of calories from total carbohydrates, and 29% of calories from proteins. All mice were maintained in a temperature‐ and light‐controlled facility with a temperature of 22 ± 2℃, humidity of 50%–60%, and a 12/12 h light‐dark cycle. They had free access to food and water ad libitum and were monitored every day for any abnormal signs like physical weakness. In addition, the weight of the mice was taken once every week to ensure the mice were in good health. All animal experiments were approved by Jeonju University Institutional Animal Care and Use Committee. They were conducted in accordance with guidelines of the institution. The mice were divided into two sets, and the extracts or compounds were administered daily for 8 weeks. The first set had 6 groups (*n* = 5 mice per group) as follows: Group 1, mice were fed with a normal diet (ND); Group 2, mice were fed with HFD; Group 3, mice were fed with HFD and orally administered with 50 mg/kg of CJE daily; Group 4, mice were fed with HFD and orally administered with 100 mg/kg of CJE daily; Group 5, mice were fed with HFD and orally administered with 200 mg/kg of CJE daily; and Group 6, mice were fed with HFD and orally administered with 100 mg/kg of MT daily. Mice in groups 1 and 2 were orally given 200 μl of distilled water daily. Mice in the second set had 5 groups (*n* = 5 mice per group) as follows: Group 1, mice were fed with normal diet (ND); Group 2, mice were fed with HFD; Group 3, mice were fed with HFD and orally administered with 0.5 mg/kg of Cir daily; Group 4, mice were fed with HFD and orally administered with 1 mg/kg of Cir daily; and Group 5, mice were fed with HFD and orally administered with 100 mg/kg of MT daily. The doses were chosen based on our laboratory standard doses for plant extract for animal experiments. Mice in groups 1 and 2 were also orally given 200 μl of distilled water daily. These mice were sacrificed by cervical dislocation.

### 2.3 Weights measurement

Body weight was measured weekly throughout the experimental period. The average difference between the initial and final weight was taken to be the average weight gain in each group. In addition, at the end of the experimental period, the mice fasted for 12 h with free access to water. After the collection of whole blood from retro‐Orbital plexus, the mice were sacrificed by cervical dislocation and the liver and epididymal fat tissues were completely excised and cleansed with saline and then weighed with an electronic balance. The tissue samples were then quickly frozen with liquid nitrogen and stored at −8℃ for subsequent use.

### 2.4 Serum biochemical Analysis

The blood samples were centrifuged at 2000 × ***g*** for 15 min at 4℃. Serum samples were collected and stored at −80℃. Assay kits were used to determine serum concentrations of AST (BioVision, Milpitas, CA, USA), ALT (BioVision), TG (BioVision), MDA (Cayman Chemical, Ann Arbor, MI, USA), and catalase (Cayman Chemical). All experiments were performed following the manufacturer's protocol. Absorbance was measured with a Tecan spectrophotometer (Tecan Group, Männedorf, Switzerland).

### 2.5 H & E staining of liver sections

After mice were sacrificed and blood samples were collected, liver tissues were collected and fixed with 10% neutral formalin for 42 h. Portions of these tissues were cut and placed in cassettes. These cassettes containing tissues were washed with three changes of phosphate‐buffered saline (PBS) for 30 min for each wash), dehydrated with a series of graded ethanol (from 60% to 100% ethanol, 30 min at each step), cleared with two changes of xylene (30 min each), and embedded with three changes of paraffin wax (1 h each). These tissues were then blocked and sectioned (5 μm thick) using a microtome. Tissue sections were stained with hematoxylin and eosin (H&E) stain (YD Diagnostics, Yongin, Korea). After washing the slides in PBS, they were dried and viewed under a light microscope (Leica, Wetzlar, Germany).

### 2.6 RNA extraction and real‐time polymerase chain reaction (RT–PCR)

Total RNAs were extracted from liver tissues using Ribospin II extraction kit (GeneAll Biotechnology, Seoul, Korea) following the manufacturer's protocol without modification. One microgram of the RNA was used to synthesize complementary DNAs using a ReverTra Ace® qPCR RT Master Mix reagent and a T100TM Bio‐Rad Thermal Cycler (Hercules, CA, USA) at 37℃ for 15 min, according to the manufacturer's instructions. PCR was performed using a SYBR Premix Ex Taq^TM^ (Takara, Shiga, Japan) and a Light Cycler (Takara). The reaction mixture for the PCR assay consisted of 0.0125 ml of TB Green Premix Ex Taq; 0.002 ml of forward and reverse primers for GAPDH (F: ggctacactgaggaccaggtsequences; R: tccaccaccctgttgctgta), COX‐2 (F: atactggaagccgagcacct; R: gtgggaggcacttgcattga), IL‐17 (F: tgtcaatgcggagggaaagc; R: ccacacccaccagcatcttc), and iNOS (F: cggagtgacggcaaacatga; R: ttccagcctaggtcgatgca); 0.002 ml of cDNA; and 0.0085 ml of distilled water. The thermal profile of the assay was as follows: 95℃ for 5 min, 40 cycles of 95℃ for 30 s and 62℃ for 30 s. All samples were run in triplicate, and the fluorescence data of target genes were analyzed with the 2−ΔΔCt method for relative quantification using GAPDH as an internal control.

### 2.7 Statistical analysis

Data presented in this study are expressed as mean ± standard deviation (*SD*). All statistical analyses were performed using SPSS statistical software version 13.0 (SPSS Inc., Chicago, IL, USA). Group means were compared using analysis of variance (ANOVA) followed by Tukey's test. p‐values <.05 were considered statistically significant.

## 3 RESULTS

### 3.1 Effects of CJE and Cirsimaritin on body weight, weight gain, liver weight, and epididymal fat weight of HFD‐induced obese mice

**FIGURE 1:**
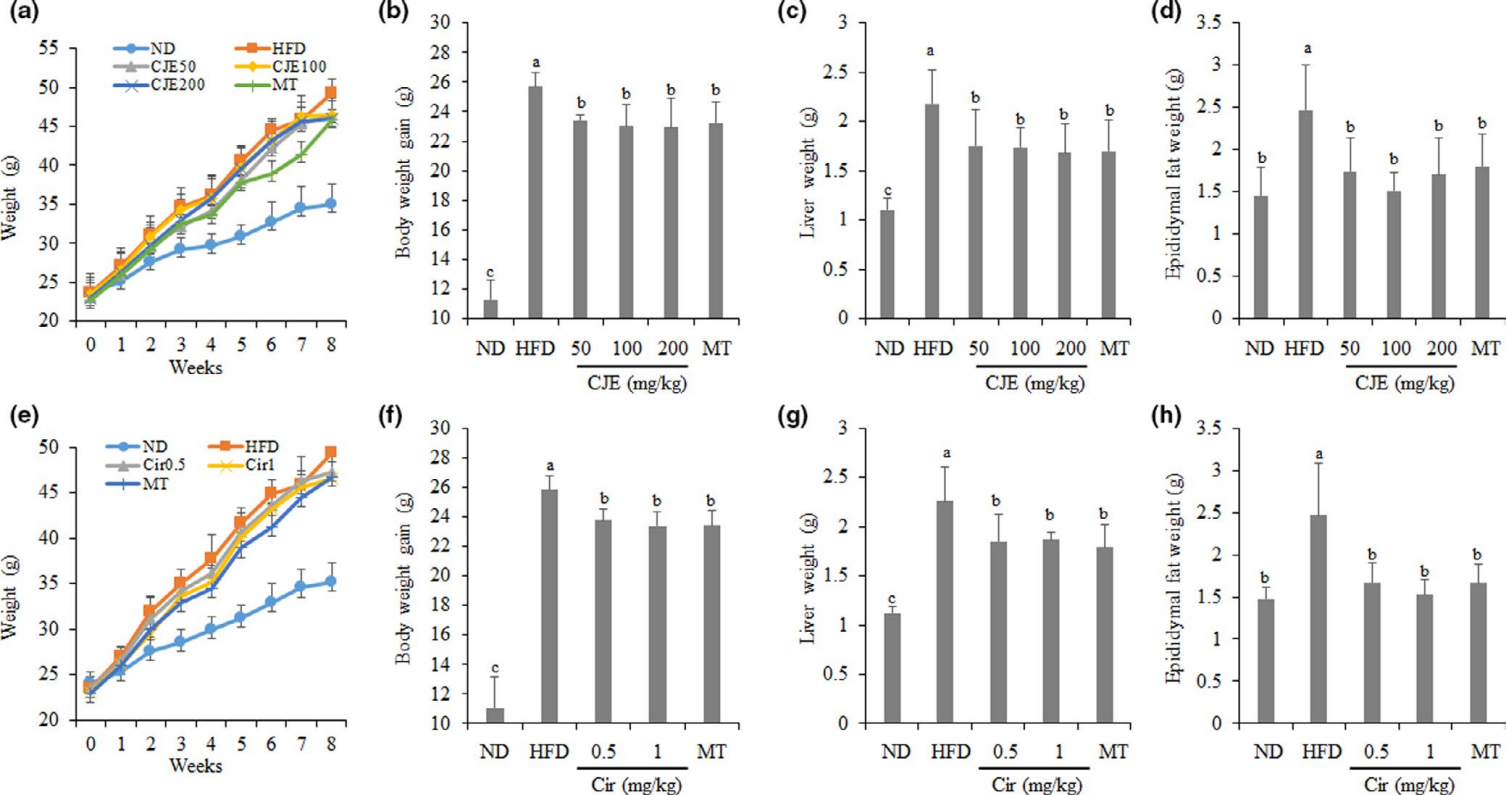
Effect of CJE and Cir on (a and e) body weight, (b and f) weight gain, (c and g) liver, and (d and h) epididymal fat weight in HFD‐induced obese mice. ND, mice (*n* = 5) were fed with a normal diet; HFD, mice (*n* = 5) were fed with high‐fat diet (HFD); HFD + CJE 50 or 100 or 200 mg/kg; HFD + MT 100 mg/kg, mice (*n* = 5 each) were fed with HFD and administered with CJE or Cir or MT at indicated concentrations. Bars with different small case letters indicate statistically significant differences between them at p < .05

As shown in Figure 1, at the start of the experiment, the initial body weights were not significantly different among the various groups. On the last day of the experiment, the final body weights of mice fed with HFD only increased significantly more than those of mice fed with ND. For mice fed with HFD and simultaneously administered with CJE at 50, 100, or 200 mg/kg and MT at 100 mg/kg, their final body weights also increased, but such increase was significantly less than that of mice fed with only HFD. However, no significant differences in final body weight were found between mice administered with different concentrations of CJE or MT. As a result, the weight gain of mice fed with HFD only was significantly higher than that of mice fed with ND. There was a significant decrease in weight gain when mice were fed with HFD and simultaneously administered with CJE at 50, 100, or 200 mg/kg and MT at 100 mg/kg, with no significant differences with respect to the CJE and MT concentration. Liver and epididymal fat weights were also found to be significantly higher than in mice fed with HFD than those of mice fed with ND. On the other hand, the liver and epididymal fat weights were found to be significantly lower for mice fed with HFD and simultaneously administered with CJE at 50, 100, or 200 mg/kg and MT at 100 mg/kg, with no significant differences with respect to the CJE and MT concentration. Similar findings were obtained when mice were fed with HFD only or with HFD and Cir at 0.5, 1 mg/kg, or MT at 100 mg/kg, showing no significant difference between groups treated with Cir and MT.

### 3.2 Effects of CJE and Cir on HFD‐induced hepatic steatosis in the liver of mice

**FIGURE 2:**
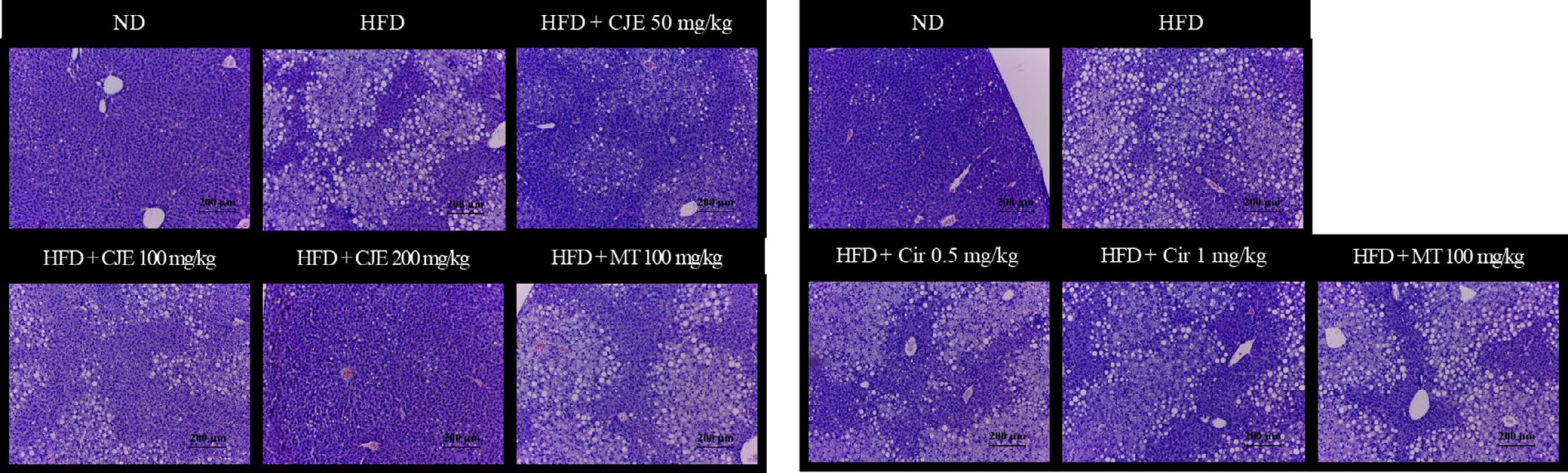
CJE and Cir prevented HFD‐induced hepatic steatosis in the liver of mice. ND, mice (*n* = 5) were fed with a normal diet; HFD, mice (*n* = 5) were fed with high‐fat diet (HFD); HFD + CJE 50 or 100 or 200 mg/kg / HFD + Cir 0.5 or 1 mg/kg / HFD + MT 100 mg/kg, mice (*n* = 5 each) were fed with HFD and administered with CJE or Cir or MT at indicated concentrations. The liver sections were stained with H&E stain to show lipid droplets in the liver

Figure 2 presents histopathological sections of the liver on the final day of the experiment. These sections show that the liver tissues of mice fed with ND had normal morphology without fat vacuoles. However, liver tissues of mice fed with HFD alone showed an abnormal morphology with severe infiltration of lipid and many fat vacuoles. The tissue morphology and fat vacuole phenomenon in mice fed with HFD and simultaneously administered with CJE at 50, 100, or 200 mg/kg, Cir at 0.5, 1 mg/kg, or MT at 100 mg/kg were improved in varying degrees. The administration of CJE at 200 mg/kg yielded better effects than other treatments. Its tissue morphology was almost similar to the tissue morphology of mice fed with ND (Figure 2).

### 3.3 Effects or CJE and Cir on serum and liver biochemical parameters

**FIGURE 3:**
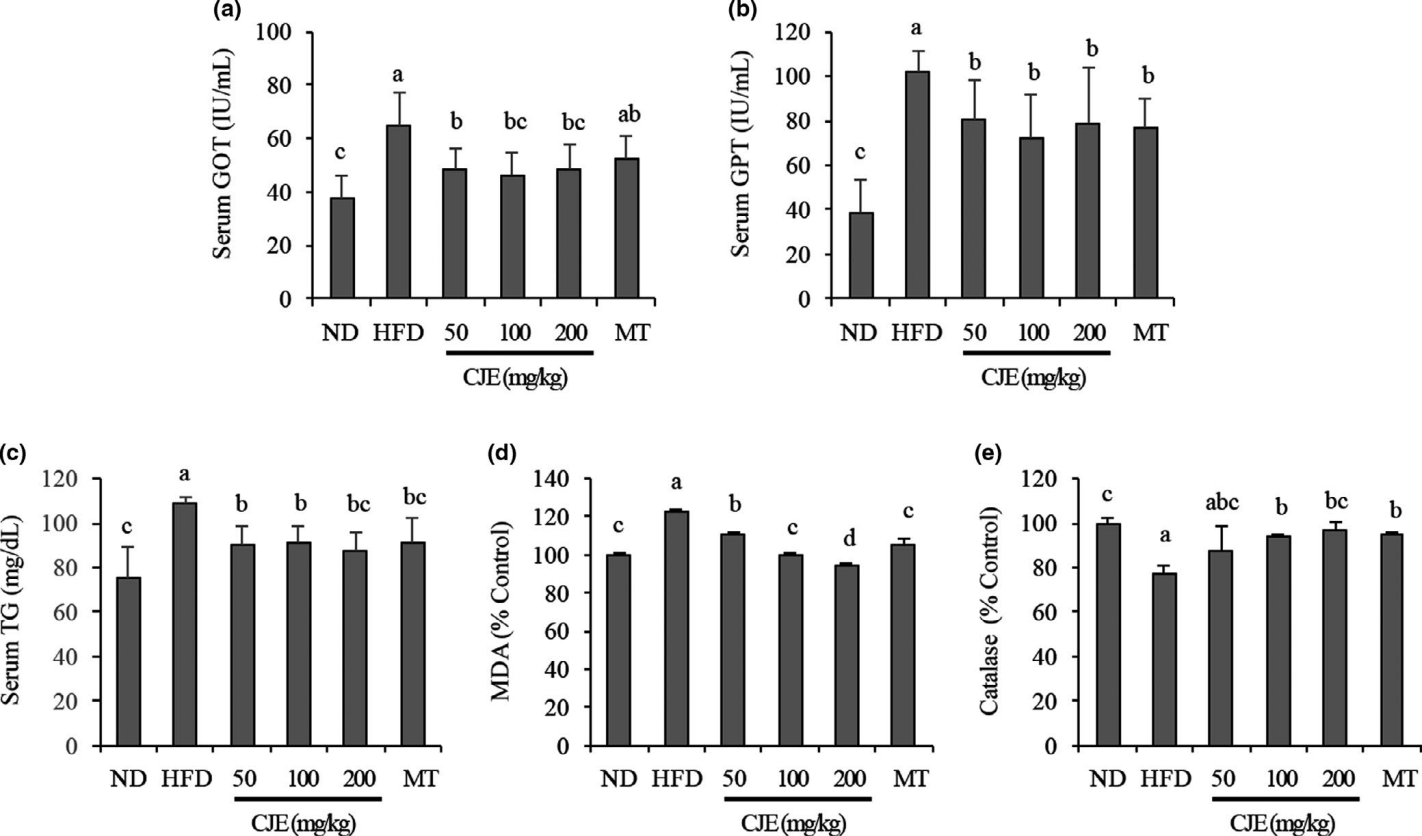
CJE decreased serum levels of AST (a) and ALT (b), liver tissue levels of TG (c) and MDA (d), and prevented catalase downregulation (e) in HFD‐fed mice. ND, mice (*n* = 5) were fed with a normal diet; HFD, mice (*n* = 5) were fed with high‐fat diet (HFD); HFD + CJE 50 or 100 or 200 mg/kg; HFD + MT 100 mg/kg, mice (*n* = 5 each) were fed with HFD and administered with CJE or Cir or MT at indicated concentrations. Bars with different small case letters indicate statistically significant differences between them at p < .05

**FIGURE 4:**
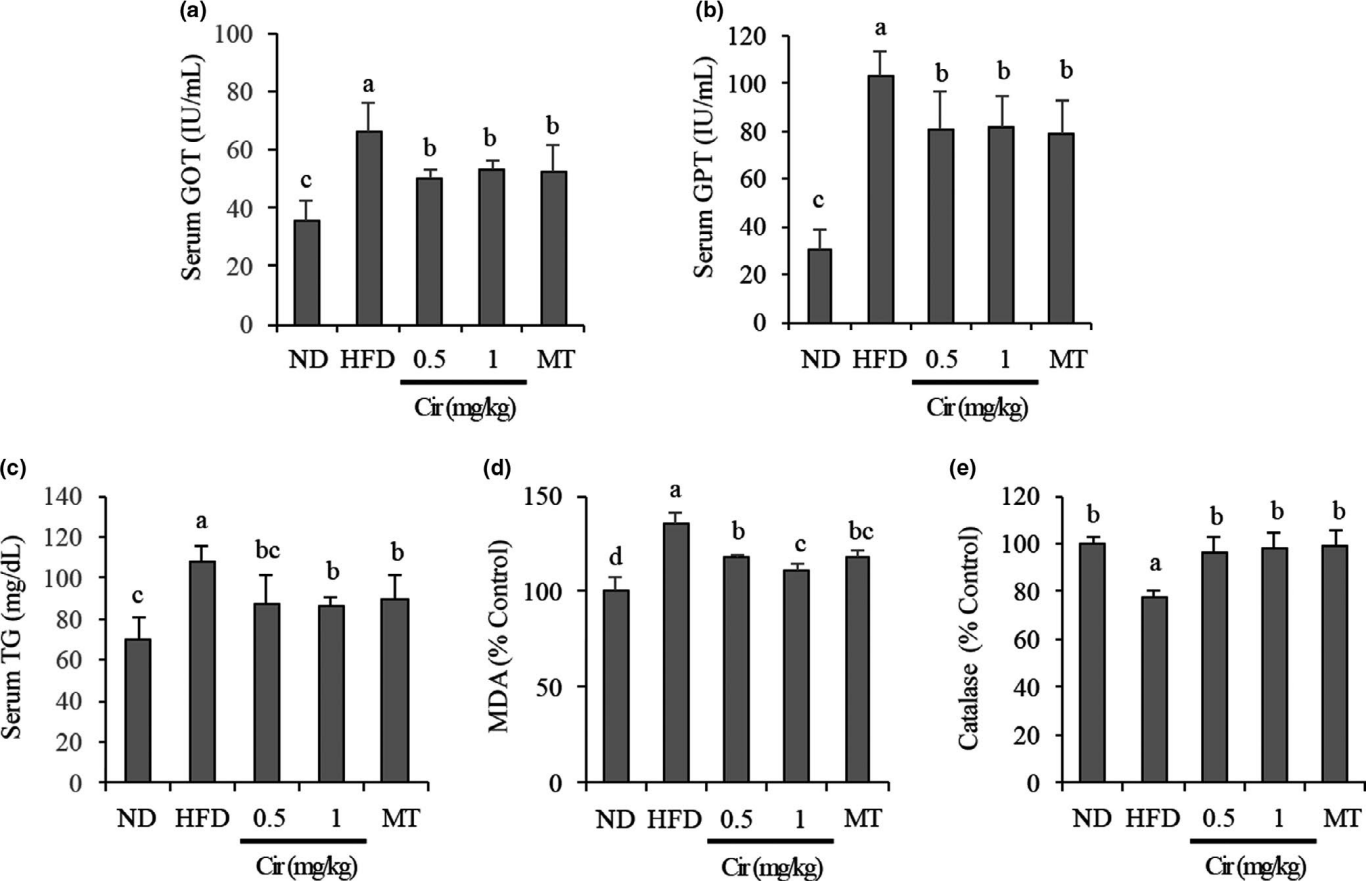
Cir decreased serum levels of AST (a) and ALT (b), liver tissue levels of TG (c) and MDA (d), and prevented catalase downregulation (e) in HFD‐fed mice. ND, mice (*n* = 5) were fed with a normal diet; HFD, mice (*n* = 5) were fed with high‐fat diet (HFD); HFD + Cir 0.5 or 1 mg/kg / HFD + MT 100 mg/kg, mice (*n* = 5 each) were fed with HFD and administered with CJE or Cir or MT at indicated concentrations. Bars with different small case letters indicate statistically significant differences between them at p < .05

Compared with mice fed with ND, the serum GOT and GPT levels in mice fed with HFD were significantly increased at the end of the experiment. GOT and GPT levels of mice fed with HFD and simultaneously administered with CJE at 50, 100, or 200 mg/kg (Figure 3a,b), Cir at 0.5, 1 mg/kg, or MT at 100 mg/kg (Figure 4a,b) were significantly reduced, showing no significant difference between treatment groups.

Figures 3 and 4 show the results of analysis of serum TG as a lipid‐related indicator. Compared with mice fed with ND, tissue levels of TG were significantly increased in mice fed with HFD alone. However, TG was significantly decreased in mice fed with HFD and simultaneously administered with CJE at 50, 100, or 200 mg/kg (Figure 3c), Cir at 0.5 or 1 mg/kg, or MT at 100 mg/kg (Figure 4c), showing no major difference between treatment groups.

The results of analysis of MDA and catalase levels in the liver are shown in Figures 3 and 4. Compared with mice fed with ND, tissue levels of MDA were significantly increased whereas catalase levels were significantly decreased in mice fed with HFD alone. However, MDA levels were decreased in mice fed with HFD and simultaneously administered with CJE at 50, 100, or 200 mg/kg (Figure 3d) or Cir at 0.5 or 1 mg/kg (Figure 4d) in a dose‐dependent manner. Mice treated with MT at 100 mg/kg also showed a significantly decreased MDA level compared with mice of the HFD‐only‐treated group. On the other hand, catalase levels in mice fed with HFD and simultaneously administered with CJE at 50, 100, or 200 mg/kg (Figure 3e), Cir at 0.5 or 1 mg/kg, or MT at 100 mg/kg (Figure 4e) were significantly increased, showing no major difference between treatment groups.

### 3.4 Effects of CJE and Cir on inflammatory related‐gene expression in the liver of mice fed with HFD

**FIGURE 5:**
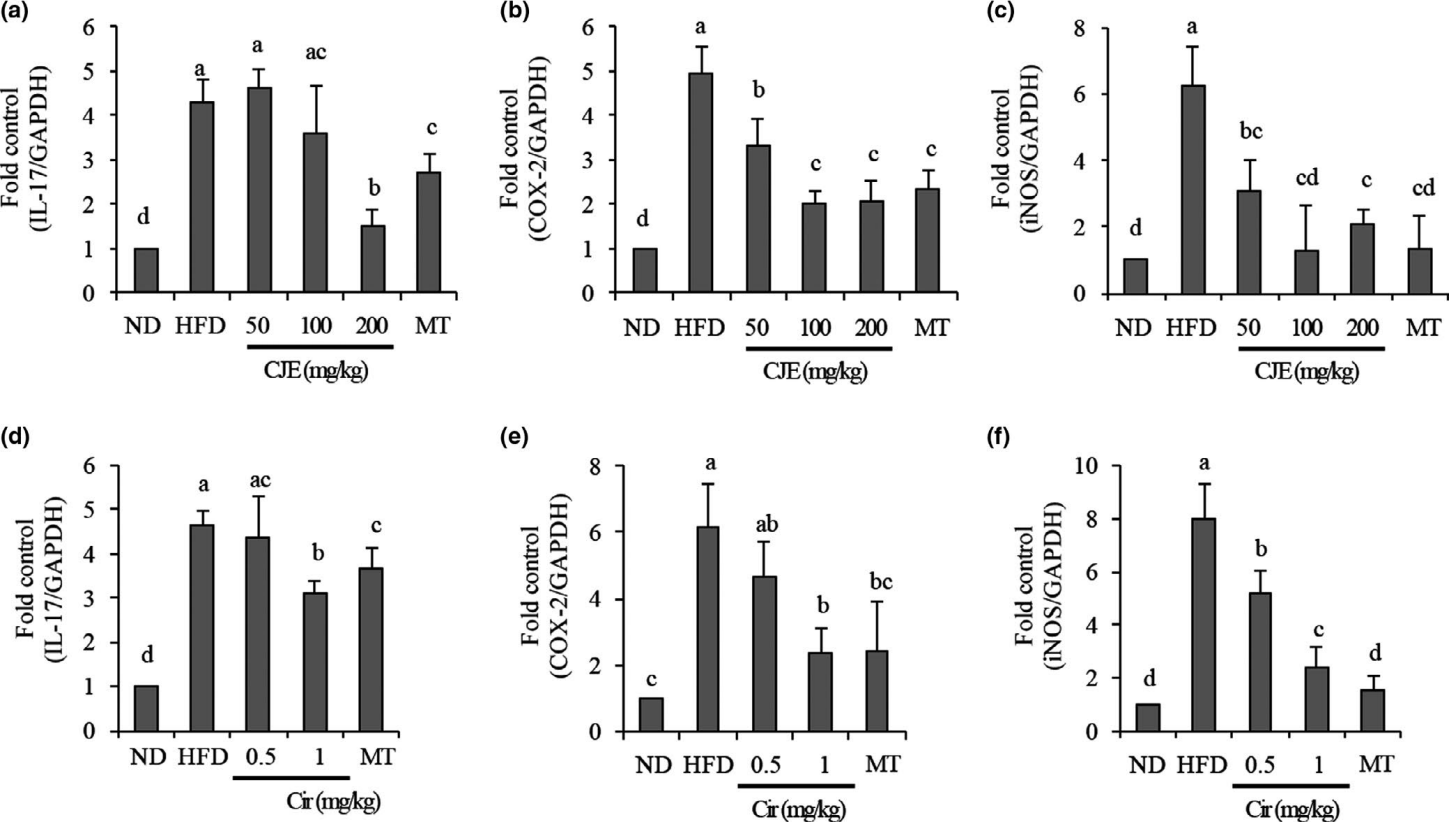
CJE and Cir prevented HFD‐induced inflammation in the liver of mice. ND, mice (*n* = 5) were fed with a normal diet; HFD, mice (*n* = 5) were fed with high‐fat diet (HFD); HFD + CJE 50 or 100 or 200 mg/kg / HFD + Cir 0.5 or 1 mg/kg / HFD + MT 100 mg/kg, mice (*n* = 5 each) were fed with HFD and administered with CJE or Cir or MT at indicated concentrations. mRNA expression for IL‐17 (a,d), COX‐2 (b,e), and iNOS (c,f) were investigated in the liver tissues. Bars with different small case letters indicate statistically significant differences between them at p < .05

Following 8 weeks of ND, HFD, CJE, or Cir administration, expression levels of IL‐17, COX‐2, and iNOS mRNAs in liver tissues of mice from various groups were measured. As shown in Figure 5, mRNA expression levels of IL‐17, COX‐2, and iNOS in mice fed with HFD alone were significantly increased than those in mice fed with ND. However, COX‐2 and iNOS mRNA expression levels in mice fed with HFD and simultaneously administered with CJE at 50, 100, or 200 mg/kg (Figure 5b,c) or Cir at 0.5 or 1 mg/kg (Figure 5e,f) were significantly decreased. IL‐17 mRNA expression was significant decreased only in mice administered with CJE at 200 mg/kg (Figure 5a), Cir at 1 mg/kg (Figure 5d), or MT at 100 mg/kg.

## 4 DISCUSSION

Changes in lifestyle, especially less exercise and frequent consumption of high‐fat‐rich food, contribute to increasing obesity‐associated chronic liver diseases such as steatosis, cirrhosis, and fibrosis (Rodríguez‐Hernández et al., 2013). These diseases are collectively called MAFLD usually characterized by excessive accumulation of fat in the liver accompanied by inflammation and hepatocellular liver injury. Inhibiting or preventing fat deposition in the liver with consumption of high‐fat diet‐rich foods will be of utmost importance for the management of MAFLD. Natural products may be promising in the prevention and treatment of chronic liver injury with fewer side effects than conventional agents. Here, we investigated effects of CJE and Cir administration in mice fed with HFD. We compared the effects of CJE and Cir with a commercial dietary supplement of Milk Thistle extract known to be effective for the treatment of MAFLD.

Generally, the consumption of HFD is associated with an increase in body weight and obesity. This was true in this study as mice fed with a high‐fat diet showed an over twofold increase in weight gain compared with mice fed with ND. Further investigation also revealed that mice fed with HFD had about two times increase in weight gain and epididymal fat weight compared to mice fed with ND. These results revealed that we had successfully induced obesity and obesity‐related fat deposition in the liver of mice. Upon administration of CJE or Cir to mice fed with HFD, we found that the final body weight of mice were significantly reduced compared to mice fed with only HFD. In addition, liver weights and epididymal fat weights of HFD‐fed mice administered with CJE or Cir were decreased compared with those of HFD‐only fed mice. Treatment of HFD‐fed mice with a hepatoprotective dietary supplement‐MT also showed similar effects as CJE and Cir. These results demonstrated that both CJE and Cir prevented general weight gain, liver weight, and epididymal fat weight increase, and hence, obesity and its related complications induced by HFD in mice. These results are similar to a previous study, showing that a variant of C. japonicum, maackii, decreased the body weights of ovariectomized rats (Park et al., 2018).

Next, we investigated effects of CJE or Cir on HFD‐induced liver damage of mice. The consumption of high‐fat diets has been associated with alteration in liver structures and hence liver damage ranging from simple steatosis to chronic liver diseases such as fibrosis and cirrhosis (Bray et al., 2004). Hepatic lipid accumulation has been suggested to be the initial pathological process in MAFLD. In this study, we observed that HFD consumption resulted in the destruction of liver architecture accompanied by fatty acid in the liver as demonstrated by an increased amount of hepatic lipid droplets in the liver. We also found that serum TG levels were increased. Thus, we had successfully induced hepatic steatosis in mice. We also confirmed reduced amounts of serum TG and lipid droplets in HFD‐fed mice administered with CJE or Cir. These results were similar to those of MT, the control plant extract. These results suggest that CJE and Cir may prevent HFD‐induced hepatic steatosis and hence fibrosis by inhibiting hepatotoxicity and accumulation of lipids in the liver. Our results are in line with another study which demonstrated that Cirsimaritin is a potent antilipogenic flavonoid that decreased fat deposition in intra‐abdominal adipose tissues of mice (Zarrouki et al., 2010). Although the precise mechanisms of action of CJE and Cir were not demonstrated in this study, previous studies have revealed that natural products could prevent liver fibrosis by inhibiting tissue inhibitors of metalloproteinases‐1 (TIMP‐1) and transforming growth factor‐β (TGF‐β1) expression in mice (Choi et al., 2017; Periasamy et al., 2014). It should be noted that sustained production of TIMPs during liver injury can inhibit the activity of collagenases, leading to reduced degradation of collagen in the liver (Henderson & Iredale, 2007). TGF‐β1 has also been observed to be a key cytokine in hepatic fibrosis that can induce the overproduction of extracellular matrix (Wu & Zern, 2000; Yoshida & Matsuzaki, 2012). Hence, we predicted that the mechanism of action of CJE and Cir in preventing fibrosis could be through its effects on TIMP‐1 and TGF‐β1 expression in the liver. Further studies are needed to ascertain this prediction.

To further understand the extent of liver damage and effects of CJE and Cir in this study, we investigated serum levels of AST and ALT in mice. AST and ALT are enzymes found in hepatocytes that are released into the blood in cases of liver damage. Thus, they can serve as indicators of liver damage. Here, we observed that serum levels of GOT and GPT were increased in mice fed with HFD, thus confirming liver damage. We also observed that serum GOT and GPT levels were reduced in HFD‐fed mice that were administered with CJE or Cir and the effects were comparable to those of MT, a control plant extract. This meant that the administration of CJE or Cir protected the liver against HFD‐induced liver damage.

Oxidative stress is a well‐established etiological factor in MAFLD and obesity. It plays a role in redox signaling that results in the activation of several major cellular detoxification pathways (Cui et al., 2013). The production of malondialdehyde (MDA) in cells is an indication of oxidative stress (Gaweł et al., 2004). We found increased levels of MDA in the liver of mice fed with HFD, obviously indicating oxidative stress in the liver. Mice fed with HFD and administered with CJE or Cir showed a decrease in MDA in the liver, indicating that CJE and Cir could protect the liver against HFD‐induced oxidative stress. Antioxidant enzymes including catalase can control cellular redox status to protect cells from oxidative damage. Therefore, we investigated the effects of CJE and Cir on catalase antioxidant enzyme expression in HFD‐fed mice to further understand the mechanism of action of CJE and Cir in ameliorating oxidative stress in the liver. We found that catalase levels in the liver were decreased in HFD‐fed mice. However, their levels were increased in the liver of mice fed with HFD and administered with CJE or Cir. These results suggest that, by preventing the downregulation of catalase in the liver, CJE and Cir can prevent HFD‐induced oxidative stress in the liver.

Oxidative stress is also known to induce hepatic inflammation and fibrosis. This occurs when antioxidant or detoxifying enzymes are depleted from the cellular environment. Patients with steatosis show the highest TNF‐α and IL‐6 levels, indicating that there is an inflammation during hepatic steatosis. Interestingly, IL‐17, a pro‐inflammatory cytokine, can exacerbate steatosis in HEPG2 cells and inflammation in MAFLD (Tang et al., 2011). Several other studies have also provided novel evidence of a critical role of IL‐17 in the progression of MAFLD, revealing that IL‐17 could accelerate high‐fat‐induced hepatic steatosis (Chackelevicius et al., 2016; Shen et al., 2017). This makes IL‐17 one of biomarkers and targets for the treatment of MAFLD. In the present study, we also found that mice fed with HFD had increased expression levels of IL‐17, COX‐2, and iNOS mRNAs in liver tissues, indicating inflammation in the liver of mice. We also observed that mice fed with HFD and treated with CJE or Cir showed decreased expression of IL‐17, COX‐2, iNOS mRNAs in the liver, with results comparable to those of MT, the control plant extract. Although not conclusive, these findings meant that CJE and Cir could prevent inflammation and hepatic steatosis, eventually preventing fibrosis in mice by downregulating the mRNA expression of pro‐inflammatory mediators in the liver.

Potential limitation to the present study includes the absence of data to show that the downregulation of the mRNA expression resulted in the decrease in the protein expression and/or secretion of IL‐17, COX‐2, iNOS. Also, the absence of NAS or SAF scoring system to quantify the pathologic changes of MAFLD is a limitation to the study.

In conclusion, CJE and its active compound Cir reduced the severity of liver injury in the experimental animal model of MAFLD by the inhibition of hepatic steatosis, oxidative stress, and inflammation. These results demonstrate that CJE and Cir have a potent preventative action against the progression of hepatic steatosis to steatohepatitis. Our study suggests that CJE and Cir might be promising agents for preventing and/or treating MAFLD. The present work serves as a pioneer study that will boost further studies on the biological potentials of this plants and its mechanism of action in preventing diseas15es.

## CONFLICT OF INTEREST

The authors declare that they have no conflict of interest.

## AUTHOR CONTRIBUTION

**Denis Nchang Che:** Data curation (lead); Formal analysis (lead); Investigation (lead); Validation (lead); Writing‐original draft (lead). **Jae Young Shin:** Data curation (lead); Formal analysis (lead); Investigation (lead); Validation (lead); Writing‐original draft (lead). **Hyun Ju Kang:** Data curation (equal); Formal analysis (equal); Investigation (equal). **Byoung Ok Cho:** Data curation (equal); Formal analysis (equal); Writing‐review & editing (equal). **Ji Hyeon Park:** Data curation (supporting); Formal analysis (supporting); Investigation (supporting). **Feng Wang:** Data curation (supporting); Formal analysis (supporting); Investigation (supporting). **Suping Hao:** Data curation (supporting); Formal analysis (supporting); Investigation (supporting). **Jae Suk Sim:** Data curation (supporting); Formal analysis (supporting); Investigation (supporting). **Dong Jun Sim:** Data curation (supporting); Formal analysis (supporting); Investigation (supporting). **Seon Il Jang:** Conceptualization (lead); Project administration (lead); Supervision (lead); Validation (lead); Writing‐review & editing (lead).

## ETHICS APPROVAL AND CONSENT TO PARTICIPATE

Mice were handled and experiments were carried out based on Jeonju University Institutional Animal Care and Use Committee guidelines with permission to carry out the experiment obtained from Jeonju University (approval no. JJU‐IACUC‐2018‐6).

## Data Availability

All the data used in this study can be made available upon reasonable request.
